# A patient safety education program in a medical physics residency

**DOI:** 10.1002/acm2.12166

**Published:** 2017-09-12

**Authors:** Eric C. Ford, Matthew Nyflot, Matthew B. Spraker, Gabrielle Kane, Kristi R. G. Hendrickson

**Affiliations:** ^1^ Department of Radiation Oncology University of Washington Seattle WA 98195 USA

**Keywords:** education, patient safety, residency program, self‐directed educational programs (SDEP)

## Abstract

Education in patient safety and quality of care is a requirement for radiation oncology residency programs according to accrediting agencies. However, recent surveys indicate that most programs lack a formal program to support this learning. The aim of this report was to address this gap and share experiences with a structured educational program on quality and safety designed specifically for medical physics therapy residencies. Five key topic areas were identified, drawn from published recommendations on safety and quality. A didactic component was developed, which includes an extensive reading list supported by a series of lectures. This was coupled with practice‐based learning which includes one project, for example, failure modes and effect analysis exercise, and also continued participation in the departmental incident learning system including a root‐cause analysis exercise. Performance was evaluated through quizzes, presentations, and reports. Over the period of 2014–2016, five medical physics residents successfully completed the program. Evaluations indicated that the residents had a positive experience. In addition to educating physics residents this program may be adapted for medical physics graduate programs or certificate programs, radiation oncology residencies, or as a self‐directed educational project for practicing physicists. Future directions might include a system that coordinates between medical training centers such as a resident exchange program.

## 1 INTRODUCTION

Patient safety and quality improvement are key components of any residency program in radiation oncology. This is well‐recognized through the ACGME requirements on quality training for physicians^1^ in place since 2006 and the CAMPEP requirements for medical physicists.^2^, ^3^ It is also reflective of trends in reimbursement models (e.g., MACRA in the US) that explicitly call for the inclusion of quality metrics.^4^ Rigorous education in quality and safety is also needed if physicians and physicists are expected to be leaders in this arena, as has been suggested.^5^, ^6^ Additionally, the recent AAPM Task Group‐100 report advocates that safety and quality “need to be incorporated in training programs for all radiation oncology disciplines.”^7^ It is also called for in other reports providing recommendations around patient safety.^8^

Although the need for quality and safety education is apparent, most residency programs currently lack a formal program for supporting this learning.^9^, ^10^ In evaluations during a one‐day safety course for physician and physics residents, only 22% of participants agreed with the statement “I have adequate education in quality improvement for my current role.”^9^ A recent survey of medical and physics residents in the US also noted that only 40% of medical physics residents agreed with the statement that “formal teaching of patient safety” was adequate in their program.^10^ Although some reports have appeared providing recommendations for a safety and quality education curriculum,^2^, ^11^ specific details are lacking and, to our knowledge, no report has yet appeared in the literature describing experiences with formal patient safety educational programs in radiation oncology residencies.

The purpose of this report was to share experience with a patient safety and quality education program developed at the University of Washington specifically for medical physics residents. While the main focus of this report is on the medical physics residency program, the experience may be useful for physician residencies and may be relevant to Maintenance of Certification through the ABR and practice accreditation programs such as APEx, in which patient safety and quality of care is a key pillar.^12^

## 2 METHODS

The patient safety and quality educational program consists of a didactic component (reading and lectures on fundamental concepts) coupled with practice‐based learning. The majority of this content is delivered in the form of a mentored rotation that is one month in duration, and there is also a component which extends throughout the residency and includes participation in the departmental incident learning program.

In designing an educational program around a broad subject like patient safety and quality, it is important to select appropriate topic areas of focus. This is particularly important in the context of a medical physics residency program. Since most programs are 24 months, the total time dedicated specifically to patient safety and quality education will necessarily be limited. In formulating focus topics we consulted the following resources: AAPM Report No. 249 on guidance for CAMPEP‐accredited residencies,^2^ the CAMPEP residency standards,^3^ the “Safety is No Accident” report from ASTRO,^12^ the report of AAPM Task Group 100,^7^ and the AAPM 2013 Summer School Proceedings on Quality and Safety.^13^ Additionally, the study of Dunscombe^8^ provides a broad summary and references to the literature, which is valuable. Table 1 shows the five topic focus areas selected for the program. The resulting topic areas are very similar to those proposed by Yeung and Greenwalt,^11^ are consistent with the recommendations from the ACGME for Pathways to Excellence,^1^ and include the specific safety‐related topics listed in the CAMPEP residency standards.^3^

**Table 1:** Topic areas for the patient safety and quality educational program. Reference materials are assigned as reading materials (c.f. Table 2)

Topic area	Learning objectives	Reference materials
Accidents and outcomes	Appreciate a few select watershed accidents in radiation oncology and the associated issues. Understand the data linking quality of treatment with outcomes.	34, 35, 36, 37, 38, 39, 40, 41, 42, 43, 44
Failure mode and effect analysis (FMEA)	Understand the FMEA formalism and become competent in its use.	7, 45, 46, 47
Incident learning and safety culture	Appreciate the various drivers of error and contributing factors. Know the various types of events, near‐misses and incidents, and how these are monitored in clinical practice. Understand the role of culture in incident learning safe practices.	25, 28, 29, 48, 49, 50, 51, 52
Principles in error proofing and quality improvement	Gain familiarity with common error‐proofing techniques and the variable effectiveness. Appreciate the difference between identifying an error (e.g., QA) and addressing the drivers of error.	53, 54, 55, 56
Quality audits	Appreciate the role of independent audits in commissioning. Gain familiarity with patient‐specific quality audits performed by physicists.	12, 57, 58, 59, 60, 61

The topic focus areas in Table 1 were grounded in a didactic component of the program that included a series of readings (see the citations in Table 1) and six lectures. A “flipped classroom” arrangement was used whereby the lectures were recorded, and the resident reviewed them in advance.^14^ This allows for a more rich discussion of the topics during in‐person meetings. These lectures and discussions were supplemented with online video lecture resources, primarily from the AAPM virtual library (aapm.org), which includes presentations from a variety of speakers (28 lectures are found between 2010 and 2015 under the keywords “patient safety”). Another resource is vimeo.com where much of the same content is available also to non‐AAPM members (e.g., search “AAPM patient safety”). Another resource (not listed in the tables below) is the Learning program on Safety and Quality the from the International Atomic Energy Agency, IAEA (https://rpop.iaea.org/RPOP/RPoP/Content/News/e-learning.htm). This course consists of 12 modules, seven of which are focused on incident learning. A certificate is available after the course is complete.

A project‐based learning component builds on and reinforces the base knowledge of concepts and content areas of the didactics. During the one‐month rotation each resident is expected to complete one project in one of the topic areas in Table 1. This often takes the form of an FMEA for a particular clinical process of the resident's choice and often involves several other department members such as dosimetrists, physicians, and therapists who are involved in the clinical workflow under study.

In addition to the rotation project, the resident participates in the incident learning program of the department. This includes attendance at the weekly safety committee meetings where near‐miss events are discussed and analyzed. The department operates a high‐volume near‐miss incident learning system where approximately 25 quality reports are analyzed every week.^15^ The resident is expected to be involved in at least one root‐cause analysis (RCA) exercise, usually motivated by findings in the incident learning system. Since reportable medical incidents are exceedingly rare in this clinic, the RCA exercise often focuses on a potentially serious near‐miss event. This has the advantage of allowing the resident to analyze a case without the time pressures and other factors associated with a reportable incident. Our experience with this approach has been positive, and we recommend that at least the first experience of a resident with RCA be focused on a near‐miss event. The expectation for RCA is decoupled from the one‐month rotation since there are often no events reported during the one‐month period that are worthy of an RCA.

Resident learning is evaluated and feedback provided in several ways: (a) A written quiz near the end of the rotation. (b) A presentation to physics faculty at the end of the rotation. These presentations are conducted at the end of every rotation in the medical physics residency and include the learning objectives for the rotation and a presentation of the rotation project. At least three physics faculty must be present and must include the rotation mentor and at least one member of the residency steering committee. (c) A written report about the rotation project. (d) A presentation of the RCA project at one of the monthly all‐staff departmental safety meetings. (e) Feedback about performance and the rotation from the mentor to the resident and also from the resident to the program director. If the resident fails to meet the educational objectives, a plan for remediation is in place which involves further study and project work. To date, however, this has not been necessary.

## 3 RESULTS

Table 2 provides an example, detailed schedule for the one‐month rotation including topic areas and assignments.

**Table 2:** Sample rotation outline including reading assignments and related lectures

Time	Topic area & associated references	Rotation project (Example here: FMEA)	Lecture
Week 1	Watershed accidents:^34^, ^35^, ^36^, ^39^, ^42^ Overview of QM chapter:^37^ (Ch 12) Harm estimates:^40^ Studies linking quality and outcomes:^38^, ^43^, ^44^ FMEA TG100:^47^ (Ch 4)	FMEA process map	1: Overview lecture 2: Watershed accidents 3: FMEA
Week 2	FMEA:^7^, ^45^, ^46^ Incident learning papers:^28^, ^48^, ^49^, ^50^, ^51^ Root‐cause analysis:^62^ National incident learning system:^25^	FMEA: collect failure modes	4: Incident learning and RCA
Week 3	Safety culture:^29^, ^52^ Error proofing:^53^ (pg. 1–13 and Ch 7) Barrier analysis:^54^ Automatic error detection:^56^	FMEA: score and rank failure modes	5: Safety culture 6: National and international incident learning systems
Week 4	Safety is no accident:^12^ (Ch 3) Safety profile assessment:^57^, ^59^ Audits and trial accreditation:^60^, ^61^ EORTC phantom experience:^58^	FMEA: interventions and rescore	7: Error Proofing and Design
Ongoing	Participation in incident learning program. Conduct at least one RCA on a near‐miss event.		

The program was developed in an iterative manner, with feedback from the residents being used to develop it into the form outlined here. One example of this was a request from the residents that they attend all weekly meetings of the departmental incident learning program starting from the beginning of the residency instead of only during the rotation since this was viewed as a rich learning environment. This is now a residency program‐level expectation. The residents start by observing these meetings and later, as their general clinical experience grows, they participate and contribute more actively to the discussion.

Over the period of 2014–2016, five medical physics residents have completed the program described here. In all cases the rotation was successfully completed according to the educational evaluations listed above. The rotation projects generally consisted of FMEA exercises on various clinical processes including: treatment planning for SBRT lung cases, the final steps of preparing a treatment plan for treatment in dosimetry, and the process for the use and approval of patient in vivo dosimetry checks. In one case the project was reported as a manuscript,^16^ and provided the opportunity to compare the results of FMEA with clinical experience by way of incident learning, which revealed some novel insights. Feedback from residents about the rotation was positive, for example, “a very useful and well‐done rotation.”

## 4 DISCUSSION

The structured educational program on quality and safety described here has been in place since 2014 and is well‐received by medical physics residents. A key feature of the program is a concentrated rotation lasting one‐month with learning objectives focused on topic areas that are important for a medical physicist in clinical practice. The program also includes continuous learning through active participation in the departmental near‐miss incident learning system.

This program is designed to address the gap in quality and safety education in residencies that has been reported in recent studies.^9^, ^10^ In one of these, a national survey of physician and physics residents, a majority of respondents reported that formal teaching of patient safety was inadequate in their program.^10^ The need for a structured learning program is also motivated by the requirements of residency accrediting bodies,^1^, ^3^ the recent trends around reimbursement models that include quality reporting components (e.g., MACRA in the USA), and the need for physicians and physicists to serve as leaders in this arena.^5^, ^6^ Training is listed as a key component in essentially all reports providing recommendations around patient safety.^8^ The program described here is also part of a broader movement to include specialized rotations in medical physics residencies and to share curricular ideas, as recently highlighted in an AAPM symposium.^17^ These specialized rotations include not only quality and safety but also clinical shadowing, introductory rotations, and other topics.

One of the strong‐points of the program described here is the focus area of incident learning and root‐cause analysis (RCA). This aspect of the program benefits from the incident learning system operated by the department.^15^ This is a high‐volume system focused on near‐miss reports that provides unique opportunities for learning^18^ and is especially valuable for residents. Incident learning and RCA are particularly important for several reasons: it is one of the pillars of safety and a requirement for practice accreditation,^12^ it is not well understood or utilized by trainees in general,^19^ and it is one of the safety topics with the largest gaps in understanding and comfort among residents within radiation oncology.^10^ A recent survey of physician and physics residents suggested that programs with an incident learning system are more effective at providing education in safety and quality.^10^ Although there may be a variety of factors accounting for this correlation, one reason is that participation in a clinical incident learning safety program engages the learner because the incidents are so relevant to radiotherapy practice.

The effectiveness of incident learning is supported by many educational theories and principles. For example, Experiential Learning, as described by Kolb^20^ requires not only hands‐on practice, but a cycle of (a) participating in a concrete experience, (b) reflective observation, (c) abstract conceptualization to make sense of the experience and finally, (d) active experimentation, where the experience is tried again, applying the new insights gained from steps 2 and 3. This cycle is remarkably similar to a Plan, Do, Study, Act (PDSA) cycle of quality improvement. Learning is very contextual, and is more effective when the learner is situated in the professional practice, and they can participate in its activities, such as the apprenticeship model of resident and fellow education. They also learn more when the content is legitimate and meaningful, as it is with RCA of safety incidents, even if they are still under supervision and still somewhat peripheral to the action.^21^ However, as trainees advance through a program, they also need to solidify their learning not just through activities and tasks, but also understanding the culture and context, which is especially important for incident learning and safety.^22^

To our knowledge, there is no report in the literature which describes the details of a formal patient safety educational program in radiation oncology residencies. Several related reports have appeared, however. Across healthcare in general there are reports about the experience of residents particularly in the realm of practice‐based quality improvement and associated metrics.^23^ Yeung and Greenwalt^11^ have proposed a framework specific to radiation oncology that would satisfy ACGME requirements for patient safety and quality improvement. The suggested topic areas are very similar to those outlined here. Fogh and Pawlicki^9^ reported on a specialized training course on quality and safety and reported substantial improvements even from this single‐day event. The University of Pennsylvania has developed a Quality and Safety Culture Education program and recently reported their five‐year experience.^24^ Though this program was developed for all staff, it did include a component specifically targeted for residents. Excellent engagement was demonstrated by physician and physics residents. There was an improvement over time for the self‐reported scores that gauged culture of safety, although the measured changes for physics residents and medical residents were not statistically significant.

A variety of challenges might be anticipated in implementing an educational program like the one described here. First, the mentor(s) might not have an extensive expertise in the topic and may feel uncomfortable teaching quality and safety concepts. This report may partially address this challenge by providing a baseline structure as well as references and background materials. It is also expected that the projects suggested here (e.g., RCA or FMEA) provide a rich opportunity for learning for both the resident and the mentor(s). These activities can also serve for Maintenance of Certification activities for the American Board of Radiology. In particular, it may provide credit for a self‐directed educational program (SDEP) which is a type of Self‐Assessment Continuing Education credit available to medical physicists (SA‐CE; see Appendix A for more details).

A second challenge might be in delivering the didactic content that supplements reading materials. The program described here employed a “flipped classroom” (i.e., recorded lectures) to deliver didactic content. This facilitates a more interactive discussion of the topics during in‐person meetings. Programs may find it challenging to develop such content. However, recorded lectures may be borrowed from colleagues or may be easily developed using low‐cost screen‐capture software such as Camtasia (TechSmith Corp., Okemos, MI, USA). The AAPM virtual library is another resource for teaching materials.

Scheduling may also represent a challenge. The program described here used a one‐month dedicated rotation. This was found to be a very compressed time scale and the rotation will be extended to 1.5 months in the future. Even with a compressed rotation, however, some residency programs may find it challenging to set aside this time from a typical 24‐month program, given the many other demands of the program. While there is no direct solution to this, it may be possible to deliver much of the learning described here outside of a rotation with a dedicated time period, such as in a weekly or monthly journal club.

A final challenge may arise if the program does not have a departmental incident learning system and, related to that, does not have a strong culture of safety. For programs which do not have a departmental incident learning system, it may be possible to review case studies from the literature or from RO‐ILS™: Radiation Oncology Incident Learning System^25^ which are publicly available.^26^ It can be expected that this will be less of an issue in the future since departmental incident learning systems are specifically called for by ASTRO and other professional societies^12^ and, in the US at least, will be incentivized by reimbursement models under the new MACRA rules. The needs of the educational program may provide an additional impetus for engaging in such a program. The tools for incident learning in radiotherapy are now well understood and described in the literature^18^, ^27^, ^28^ and specialty‐specific systems are available, such as the RO‐ILS system^25^ sponsored by ASTRO and AAPM and the Center for Assessment of Radiological Sciences PSO system (cars‐pso.org). Related to this is the need for a strong culture of safety, which is a driving force for quality care. Culture of safety has been linked to patient outcomes^29^ and has been specifically mentioned in the radiation oncology context.^5^, ^24^

While this report has focused on medical physics residents, parts of the program described here may have a broader applicability. Parts of the program may be adapted to serve the continuing medical education needs of faculty and staff (see Appendix A). It may also be possible to adapt the program for physician residents, but changes would need to be made including a modification of the topic focus areas (e.g., Table 1). For instance, incident learning should include the issue of error disclosure and professional aspects.^30^ Principles of error proofing (Table 1 & 2, for example, barrier analysis, QA, and automatic error detection) could be modified to have less of a physics focus, and quality audits could be modified to focus less on commissioning and phantoms and more on issues like physician peer review.^31^, ^32^ Adapting aspects of the curriculum may also be of interest to graduate or certificate program directors.

There are some limitations to this study. One important limitation is that not all safety‐related topics can be included in an educational program like the one presented here, and Table 1 should not be taken as an exhaustive list. For example, one topic which is not included is statistical process control. This technique, which has attracted attention recently, involves analyzing and tracking the results of some procedure or test over time to establish clinical action levels^33^ and has an analog with physician‐reported performance metrics, which have been called for in the context of trainee education.^23^ While such topics are called for in some reports,^11^ it must be acknowledged that not all aspects of patient safety and quality of care can be included in a relatively focused educational program like the one described here.

The program described here is expected to evolve and develop further. One future direction might be a distributed program that is coordinated between a few centers. This might include a combined teaching component, which leverages the expertise of faculty at various centers, and/or a resident exchange program which facilitates a shared learning experience. Another direction may be to encourage partnerships between physician and physics residents to conduct projects together. Any future effort would benefit from a certification process such as that eLearning course from the IAEA (https://rpop.iaea.org/RPOP/RPoP/Content/News/e-learning.htm).

## CONFLICT OF INTEREST

The authors declare no conflict of interest.

## APPENDIX A

### MAINTENANCE OF CERTIFICATION CREDITS FOR MEDICAL PHYSICISTS UNDER THE SDEP MECHANISM

*Summary of SDEP mechanism*: Self‐directed educational programs (SDEP) are one method that the American Board of Radiology (ABR) recognizes for Medical Physicist for fulfilling Part 2 of maintenance of certification (MOC) requirements (i.e., Lifelong Learning and Self‐Assessment). Current ABR requirements call for 75 Category 1 CE credits in a three‐year period. At least 25 of these credits must be Self‐Assessment CE (SA‐CE). One common type of SA‐CE is ABR‐prequalified SAMs credits offered at meetings and elsewhere. Another type of SA‐CE credit specifically available to medical physicists is the SDEP. Details are found at www.theabr.org/moc-rp-comp2: “A maximum of one SDEP may be recorded yearly. Fifteen CE credits are given for each completed SDEP, and project samples can be found on the ABR web−site.”

The template provides an example for SDEP project related to the educational program described in this study.

**SDEP project for ABR MOC credit**

**Title:** Develop a patient safety and education program for medical physics residents

**Category:** Education

**Date Initiated:** TBD

**Date Completed:** TBD

### A SIGNIFICANCE

Patient safety and quality of care are key core concepts for medical physicists and are a competency of medical physics residents required by CAMPEP. As future leaders it is essential that residents have a strong foundation in the concepts and practice of safety and quality. The program developed here will support these educational needs.

### B APPROACH

*Example*: I will develop the structure for an educational program for residents in our medical physics program. This will consist of a one‐month rotation. The rotation will include a series of background readings on quality and safety. In this SDEP I will also develop a series of four lectures to support this learning. A future SDEP project (planned for next year) is to develop a rotation project centering on failure mode and effects analysis (FMEA) which will allow the resident to experience hands‐on learning.

### C EVALUATION OF ACHIEVEMENT

#### 1 Prospective statement (provided when SDEP is initiated)

*Example*: I will assemble a list of readings and review them myself in detail. I will develop and deliver four power‐point lectures. At the end of the rotation, residents will provide an evaluation of the rotation which will help assess the success of this project.

#### 2 Final statement (provided when SDEP is completed)

*Example*: The project has resulted in a finalized reading list of 30 key studies, three book chapters and four educational videos from the AAPM virtual library (see list below). These are assigned to the resident when they start the rotation. I have also developed and delivered four power‐point presentations on the following topics: “Example errors in radiation oncology,” “QA — What does it mean?”, “Root‐cause analysis and how to investigate an error” and “Culture of Safety: Incident learning and me.” Resident assessments have been positive. One resident, for example, noted: “This is better than quantum physics II. I loved it!”

### D IMPACT ON PRACTICE/OUTCOME STATEMENT

#### 1 Prospective statement (provided when SDEP is initiated)

*Example*: The project addresses the important need for a structured program in quality and safety for radiation oncology physics residents. This is expected to have a significant impact on their understanding as they move toward more independent practice.

#### 2 Final statement (provided when SDEP is completed)

*Example*: The implementation of this program has helped residents to better understand key concepts in patient safety and quality of care. They have mastered the key concepts and have a basic facility with some of the tools for quality management. The program has also provided an opportunity for mentors to further learn these topics in detail.

## References

[1] Education ACfGM . Clinical Learning Environment Review (CLER): CLER Pathways to Excellence. Chicago, IL: Accreditation Council for Graduate Medical Education; 2014.

[2] AAPM . Essentials and guidelines for clinical medical physics residency training programs, AAPM Report No. 249. College Park, MD; 2013. 10.1120/jacmp.v15i3.4763 PMC5711071 24892354

[3] CAMPEP . Standards for Accreditation of Residency Educational Programs in Medical Physics. Alexandria, VA: Commission on Accreditation of Medical Physics Educational Programs (CAMPEP) Inc.; 2016.

[4] ASTRO . Medicare access and CHIP reauthorization act of 2015 (MACRA); 2016 www.astro.org/macra.

[5] Hendee WR , Herman MG . Improving patient safety in radiation oncology. Med Phys. 2011;38:78–82. 21361177 10.1118/1.3522875

[6] Marks LB , Rose CM , Hayman JA , Williams TR . The need for physician leadership in creating a culture of safety. Int J Radiat Oncol Biol Phys. 2011;79:1287–1289. 21277115 10.1016/j.ijrobp.2010.12.004

[7] Huq MS , Fraass BA , Dunscombe PB , et al. The report of Task Group 100 of the AAPM: application of risk analysis methods to radiation therapy quality management. Med Phys. Jul 2016;43:4209. 27370140 10.1118/1.4947547 PMC4985013

[8] Dunscombe P . Recommendations for safer radiotherapy: what's the message? Front Oncol. 2012;2:129. 23061045 10.3389/fonc.2012.00129 PMC3460278

[9] Fogh S , Pawlicki T . A report on quality and safety education for radiation oncology residents. Int J Radiat Oncol Biol Phys. 2014;90:988–989. 25539363 10.1016/j.ijrobp.2014.08.009

[10] Spraker M , Nyflot M , Hendrickson K , Ford E , Kane G , Zeng J . A survey of residents’ experience with patient safety and quality improvement concepts in radiation oncology. Pract Radiat Oncol. 2017;7:e253–e259. 28462894 10.1016/j.prro.2016.11.008

[11] Yeung A , Greenwalt J . A framework for quality improvement and patient safety education in radiation oncology residency programs. Pract Radiat Oncol. 2015;5:423–426. 26432678 10.1016/j.prro.2015.07.008

[12] Zeitman A , Palta J , Steinberg M . Safety is No Accident: A Framework for Quality Radiation Oncology and Care; 2012.

[13] Thomadsen B, Dunscombe P, Ford E, Huq MS, Pawlicki T, Sutlief S, eds. Quality and Safety in Radiotherapy: Learning the New Approaches in Task Group 100 and Beyond. Madison, WI: Medical Physics Publishing; 2013.

[14] Prober CG , Khan S . Medical education reimagined: a call to action. Acad Med. Oct 2013;88:1407–1410. 23969367 10.1097/ACM.0b013e3182a368bd

[15] Nyflot MJ , Zeng J , Kusano AS , et al. Metrics of success: measuring impact of a departmental near‐miss incident learning system. Pract Radiat Oncol. 2015;5:e409–416. 26231595 10.1016/j.prro.2015.05.009

[16] Yang F , Cao N , Young L , et al. Validating FMEA output against incident learning data: a study in stereotactic body radiation therapy. Med Phys. Jun 2015;42:2777–2785. 26127030 10.1118/1.4919440

[17] Hendrickson K . Excellence in medical physics residency education. AAPM Annual Meeting, Washington, DC; 2016.

[18] Mutic S , Brame RS , Oddiraju S , et al. Event (error and near‐miss) reporting and learning system for process improvement in radiation oncology. Med Phys. 2010;37:5027–5036. 20964222 10.1118/1.3471377

[19] Logio LS , Ramanujam R . Medical trainees’ formal and informal incident reporting across a five‐hospital academic medical center. Jt Comm J Qual Patient Saf.Jan 2010;36:36–42. 20112664 10.1016/s1553-7250(10)36007-7

[20] Kolb DA . Experiential Learning: Experience as the Source of Learning and Development. Englewood Cliffs, NJ: Prentice‐Hall; 1984.

[21] Lave J , Wenger E . Situated Learning: Legitimate Peripheral Participation. Cambridge England; New York: Cambridge University Press; 1991.

[22] Brown JS , Collins A , Duguid P . Situated cognition and the culture of learning. Educ Research. 1989;18:32–42.

[23] Patow CA , Karpovich K , Riesenberg LA , et al. Residents’ engagement in quality improvement: a systematic review of the literature. Acad Med. Dec 2009;84:1757–1764. 19940586 10.1097/ACM.0b013e3181bf53ab

[24] Woodhouse KD , Volz E , Bellerive M , et al. The implementation and assessment of a quality and safety culture education program in a large radiation oncology department. Pract Radiat Oncol. 2016;6:e127–134. 26850651 10.1016/j.prro.2015.11.011

[25] Hoopes DJ , Dicker AP , Eads NL , et al. RO‐ILS: radiation oncology incident learning system: a report from the first year of experience. Pract Radiat Oncol. 2015;5:312–318. 26362705 10.1016/j.prro.2015.06.009

[26] ASTRO . RO‐ILS: radiation oncology incident learning system; 2016 https://www.astro.org/RO-ILS-Education.aspx 10.1016/j.prro.2015.06.009 26362705

[27] Clark BG , Brown RJ , Ploquin J , Dunscombe P . Patient safety improvements in radiation treatment through 5 years of incident learning. Pract Radiat Oncol. 2013;3:157–163. 24674359 10.1016/j.prro.2012.08.001

[28] Ford EC , de Fong Los Santos L , Pawlicki T , Sutlief S , Dunscombe P. Consensus recommendations for incident learning database structures in radiation oncology. Med Phys. 2012;39:7272–7290. 23231278 10.1118/1.4764914

[29] Mardon RE , Khanna K , Sorra J , Dyer N , Famolaro T . Exploring relationships between hospital patient safety culture and adverse events. J Patient Saf. Dec 2010;6:226–232. 21099551 10.1097/PTS.0b013e3181fd1a00

[30] Evans SB , Yu JB , Chagpar A . How radiation oncologists would disclose errors: results of a survey of radiation oncologists and trainees. Int J Radiat Oncol Biol Phys. 2012;84:e131–137. 22560545 10.1016/j.ijrobp.2012.03.010

[31] Hoopes DJ , Johnstone PA , Chapin PS , et al. Practice patterns for peer review in radiation oncology. Pract Radiat Oncol. 2015;5:32–38. 25413419 10.1016/j.prro.2014.04.004

[32] Marks LB , Adams RD , Pawlicki T , et al. Enhancing the role of case‐oriented peer review to improve quality and safety in radiation oncology: executive summary. Pract Radiat Oncol. Jul 2013;3:149–156. 24175002 10.1016/j.prro.2012.11.010 PMC3808744

[33] Pawlicki T , Whitaker M , Boyer AL . Statistical process control for radiotherapy quality assurance. Med Phys. Sep 2005;32:2777–2786. 16266091 10.1118/1.2001209

[34] Bogdanich W . Radiation Offers New Cures, and Ways to do Harm. New York, NY: New York Times; 2010, 1.

[35] Bogdanich W , Ruiz RR . Radiation Errors Reported in Missouri. New York, NY: New York Times; 2010, A17.

[36] CoxHealth . CoxHealth announces some BrainLAB stereotactic radiation therapy patients received increased radiation dose; 2010 http://www.coxhealth.com/body.cfm?id=3701.

[37] Dieterich S , Ford E , Pavord D , Zeng J . Quality and safety improvement in radiation oncology. Practical Radiation Oncology Physics. Philadelphia, PA: Elsevier; 2016:165–178.

[38] Engels B , Soete G , Verellen D , Storme G . Conformal arc radiotherapy for prostate cancer: increased biochemical failure in patients with distended rectum on the planning computed tomogram despite image guidance by implanted markers. Int J Radiat Oncol Biol Phys. 2009;74:388–391. 19056185 10.1016/j.ijrobp.2008.08.007

[39] Executive TS . Unintended Overexposure of Patient Lisa Norris During Radiotherapy Treatment at the Beatson Oncology Centre, Glasgow in January 2006. Edinburgh: The Scottish Executive; 2006.

[40] Ford EC , Terezakis S . How safe is safe? Risk in radiotherapy. Int J Radiat Oncol Biol Phys. 2010;78:321. 20832662 10.1016/j.ijrobp.2010.04.047

[41] IAEA . IAEA training materials: module 2, prevention of accidental exposure in radiotherapy. Vienna: International Atomic Energy Agency; 2009.

[42] Leveson NG , Turner CS . An investigation of the Therac‐25 accidents. Computer. 1993;26:18–41.

[43] Ohri N , Shen XL , Dicker AP , Doyle LA , Harrison AS , Showalter TN . Radiotherapy protocol deviations and clinical outcomes: a meta‐analysis of cooperative group clinical trials. Jnci‐J Natl Cancer I.Mar 2013;105:387–393. 10.1093/jnci/djt001 PMC3601950 23468460

[44] Peters LJ , O'Sullivan B , Giralt J , et al. Critical impact of radiotherapy protocol compliance and quality in the treatment of advanced head and neck cancer: results from TROG 02.02. J Clin Oncol. 2010;28:2996–3001. 20479390 10.1200/JCO.2009.27.4498

[45] Ford EC , Gaudette R , Myers L , et al. Evaluation of safety in a radiation oncology setting using failure mode and effects analysis. Int J Radiat Oncol Biol Phys. 2009;74:852–858. 19409731 10.1016/j.ijrobp.2008.10.038 PMC3655406

[46] Ford EC , Smith K , Terezakis S , et al. A streamlined failure mode and effects analysis. Med Phys. Jun 2014;41:061709. 24877804 10.1118/1.4875687

[47] Thomadsen B , Dunscombe P , Ford E , Huq MS , Pawlicki T , Sutlief S . Risk Assessment Using the TG‐100 Methodology. Quality and Safety in Radiotherapy: Learning the New Approaches in Task Group 100 and Beyond. Madison, WI: Medical Physics Publishing; 2013.

[48] Bissonnette JP , Medlam G . Trend analysis of radiation therapy incidents over seven years. Radiother Oncol. 2010;96:139–144. 20542343 10.1016/j.radonc.2010.05.002

[49] Clark BG , Brown RJ , Ploquin JL , Kind AL , Grimard L . The management of radiation treatment error through incident learning. Radiother Oncol. 2010;95:344–349. 20400189 10.1016/j.radonc.2010.03.022

[50] Cooke D , Dubetz M , Heshmati R , et al. A Reference Guide for Learning from Incidents in Radiation Treatment. Edmonton: Alberta Heritage Foundation for Medical Research; 2006.

[51] Cunningham J , Coffey M , Knöös T , Holmberg O . Radiation oncology safety information system (ROSIS) – Profiles of participants and the first 1074 incident reports. Radiother Oncol. 2010;97:601–607. 21087801 10.1016/j.radonc.2010.10.023

[52] Kusano AS , Nyflot MJ , Zeng J , et al. Measurable improvement in patient safety culture: a departmental experience with incident learning. Pract Radiat Oncol. 2015;5:e229–237. 25413404 10.1016/j.prro.2014.07.002

[53] AHRQ AfHRaQ . Mistake‐Proofing the Design of Health Care Processes. Rockville, MD: Agency for Healthcare Research and Quality (AHRQ), AHRQ Publication No. 07‐0020; 2007.

[54] Ford EC , Terezakis S , Souranis A , Harris K , Gay H , Mutic S . Quality control quantification (QCQ): a tool to measure the value of quality control checks in radiation oncology. Int J Radiat Oncol Biol Phys. 2012;84:e263–269. 22682808 10.1016/j.ijrobp.2012.04.036

[55] Grout JR . Mistake proofing: changing designs to reduce error. Qual Saf Health Care. 2006;15(SUPPL. 1):i44–i49. 17142609 10.1136/qshc.2005.016030 PMC2464876

[56] Kalet AM , Gennari JH , Ford EC , Phillips MH . Bayesian network models for error detection in radiotherapy plans. Phys Med Biol. 2015;60:2735–2749. 25768885 10.1088/0031-9155/60/7/2735

[57] Dunscombe P , Brown D , Donaldson H , et al. Safety profile assessment: an online tool to gauge safety‐critical performance in radiation oncology. Pract Radiat Oncol. 2015;5:127–134. 25748005 10.1016/j.prro.2014.10.012

[58] Fairchild A , Collette L , Hurkmans CW , et al. Do results of the EORTC dummy run predict quality of radiotherapy delivered within multicentre clinical trials? Eur J Cancer. Nov 2012;48:3232–3239. 22766515 10.1016/j.ejca.2012.06.002

[59] Ford EC , Brown D , Donaldson H , et al. Patterns of practice for safety‐critical processes in radiation oncology in the United States from the AAPM safety profile assessment survey. Pract Radiat Oncol. 2015;5:e423–429. 26231597 10.1016/j.prro.2015.06.005

[60] Ibbott G , Ma CM , Rogers DW , Seltzer SM , Williamson JF . Anniversary paper: fifty years of AAPM involvement in radiation dosimetry. Med Phys. Apr 2008;35:1418–1427. 18491537 10.1118/1.2868765

[61] Kirby TH , Hanson WF , Gastorf RJ , Chu CH , Shalek RJ . Mailable TLD system for photon and electron therapy beams. Int J Radiat Oncol Biol Phys. Feb 1986;12:261–265. 3949577 10.1016/0360-3016(86)90107-0

[62] Vincent C , Taylor‐Adams S , Chapman EJ , et al. How to investigate and analyse clinical incidents: clinical risk unit and association of litigation and risk management protocol. BMJ. 2000;320:777–781. 10720366 10.1136/bmj.320.7237.777 PMC1117773

